# The roles of autophagy and thyroid hormone in the pathogenesis and treatment of NAFLD

**DOI:** 10.20517/2394-5079.2021.82

**Published:** 2021-11-05

**Authors:** Jin Zhou, Rohit A. Sinha, Paul M. Yen

**Affiliations:** 1 Program of Cardiovascular and Metabolic Disorders, Duke-NUS Medical School, Singapore 169857, Singapore; 2 Department of Endocrinology, Sanjay Gandhi Postgraduate Institute of Medical Sciences, Lucknow 226014, India; 3 Department of Medicine Duke Molecular Physiology Institute, Duke University Medical Center, Durham, NC 27710, USA

**Keywords:** Autophagy, mitophagy, thyroid hormone, lipid oxidation, NAFLD

## Abstract

Non-alcoholic fatty liver disease (NAFLD) is the most prevalent chronic liver disorder worldwide. It comprises simple steatosis and non-alcoholic steatohepatitis (NASH), which can further progress to cirrhosis and hepatocellular carcinoma. The pathogenesis of NAFLD involves genetic, environmental, and endocrine factors, and several molecular mechanisms have been identified. In this review, we discuss the recent findings on the role of autophagy, in particular lipophagy and mitophagy, in hepatic lipid oxidation. We discuss the pre-clinical and clinical evidence suggesting that impairment of autophagy exacerbates NAFLD progression and restoration of autophagy exerts beneficial effects on NAFLD. We discuss how thyroid hormone (TH) simultaneously regulates lipophagy, mitophagy, and mitochondrial biogenesis to increase β-oxidation of fatty acids and reduce steatosis in the liver. Lastly, we discuss the recent clinical progress in using TH or thyromimetics in treating NAFLD/NASH.

## Introduction

Non-alcoholic fatty liver disease (NAFLD) is the most prevalent chronic liver disease. Its earliest manifestation is non-alcoholic fatty liver (NAFL) occurring in the absence of significant consumption of alcohol or other known causes of liver steatosis such as viral hepatitis and medication (e.g., tamoxifen, amiodarone, and methotrexate)^[1]^. NAFL can progress to non-alcoholic steatohepatitis (NASH), which is characterized by progressive inflammation, hepatocyte death, and fibrosis^[2]^. The global prevalence of NAFLD is estimated to be 20%-40%^[3,4]^, while the prevalence of NASH ranges between 1.5% and 6.45%^[5]^ and is expected to rise over the next 20 years^[6]^. NASH now represents the fastest growing indication for liver transplantation in Western countries^[7]^.

NAFLD is highly associated with features of metabolic syndrome such as diabetes, obesity, and dyslipidemia. The presence of NASH and fibrosis are also strongly associated with increased risk of cardiomyopathy and arrhythmias, chronic kidney disease, sarcopenia, and other extrahepatic malignancies^[8–10]^. In addition, biopsy-confirmed fibrosis is associated with increased risk of mortality and liver-related morbidity in patients with NAFLD^[11]^. Currently, lifestyle modifications such as diet and exercise play important roles in the management of NASH since there are no FDA-approved drugs. However, several compounds now are undergoing Phase 3 clinical trials in patients with NASH, including farnesoid X receptor (FXR) agonist obeticholic acid (REGENERATE/NCT02548351), stearoyl-CoA desaturase 1 (SCD1) inhibitor aramchol (NCT04104321), C-C chemokine receptors type 2 (CCR2) and 5 (CCR5) dual antagonist cenicriviroc (AURORA/NCT03028740), glucagon-like peptide 1 (GLP-1) receptor agonist semaglutide (NCT04822181), and selective thyroid hormone receptor β (THRβ) agonist MGL-3196 (MAESTRO-NASH/NCT03900429).

NAFLD is a chronic and complex disease, and it has been postulated that “multiple parallel hits” may be involved in the molecular pathogenesis of NAFLD. There also are metabolic conditions associated with NAFLD/NASH (e.g., diabetes and hypothyroidism) which may predispose, or even play a pathogenic role, in NAFLD. In this review, we discuss the current controversy regarding the change in nomenclature of NAFLD to MAFLD to reflect the key role of metabolism in this condition. We also discuss the impairment of the autophagy in NAFLD, with a focus on the roles of lipophagy and mitophagy and their potential as “targets” to reduce steatosis by promoting lipid oxidation in NAFLD, particularly by thyroid hormone (TH). Finally, we review the clinical studies thus far supporting TH and its analogs as novel pharmacological agents for NAFLD/NASH treatment.

## NAFLD vs. MAFLD controversy

Recently, it has been proposed that the old nomenclature “NAFLD” be replaced by metabolic dysfunction-associated fatty liver disease (MAFLD)^[12,13]^. In this new system, the diagnosis of MAFLD is based on the presence of hepatic steatosis, in addition to one of the following three criteria: (1) obesity; (2) presence of Type 2 diabetes mellitus (T2DM); or (3) evidence of metabolic dysregulation^[12,13]^. It has been suggested that the new nomenclature and diagnostic criteria reflect the pathogenesis more accurately and emphasize the tight connection between this condition and metabolic dysfunction. However, the proposal to rename NAFLD to MAFLD still is controversial. Although MAFLD reflects some of the relevant risk factors for this liver disease, it has shortcomings since it does not distinguish between metabolic defects that have an etiological role for MAFLD and those that are consequences of it^[14]^. While the umbrella term “MAFLD” still covers a spectrum of disease stages, it lacks clear staging criteria to categorize disease severity making it difficult to employ this classification system to assess clinical progression. In addition, the elimination of “NASH” as a subtype could place several current Phase 2b and 3 trails in jeopardy since these trials were designed using the old criteria for NASH to assess drug efficacy. Thus, without better understanding of the pathogenesis of NAFLD and improvements in classifying the patients, simply renaming the disease may not accelerate the discovery of biomarkers or drug development, even if national drug regulatory agencies decide to adopt the new nomenclature.

Recently, several other suggestions for a better classification of patients with NAFLD have been proposed. Singh *et al*.^[15]^ proposed a “MEGA-D” classification of NAFLD in which NAFLD remains the umbrella entity and has new subgroups under it such as NAFLD-M (metabolic syndrome-associated NAFLD), NAFLD-E (environmental stressor-related NAFLD), NAFLD-G (genetic factor-associated NAFLD), NAFLD-A (bile acid dysregulation-related NAFLD), and NAFLD-D (gut dysbiosis-related NAFLD). Lonardo *et al*.^[16]^ proposed a “LED” classification in which the prefix “L” is for “liver” which mainly identifies the liver histological determinants, “E” is for common “extra-hepatic” manifestations such as metabolic and cardiovascular profiles, and “D” is for “determinants” in individual patients such as sex, menopause, and metabolic syndrome status, which may have epigenetic roles, and specific single nucleotide polymorphisms that could have genetic implications.

Currently, within the hepatology field, it is difficult to resolve the controversies surrounding the use of “NAFLD” *vs*. “MAFLD” or other proposed nomenclatures that try to consider and incorporate other key features of the liver disease within their terminology. Adopting new nomenclature is complicated further by the fact this field is rapidly advancing with new information on the genetic, metabolic, and cellular mechanisms of NAFLD. It would be appropriate and timely for an international consensus group comprised of academic, pharmaceutical, and regulatory members to decide whether a new nomenclature is warranted at this time and to agree upon the precise meanings of any new terminology that is adopted. In this review, we use NAFLD or NASH since they are still the most commonly used terms in the literature.

## Autophagy in NAFLD

### Autophagy: the process

Macroautophagy, referred as autophagy hereafter, is a highly conserved autophagosome- and lysosome-dependent degradation process. Autophagy is essential for cellular function during normal physiological conditions and is further induced as an adaptive response to different stresses such as starvation or hypoxia. Autophagy begins by the formation of cup-shaped structures called “phagophores” that originate from specific endoplasmic reticulum (ER) membrane domains called “phagophore assembly sites (PASs)”. Interestingly, in addition to giving rise to phagophores, PASs can also serve as membrane contact sites that generate nascent ER-mitochondria and ER-plasma membrane structures^[17]^. During the elongation and expansion of phagophores into spheres, cytoplasmic materials and damaged organelle remnants become autophagic cargo engulfed within the inner membrane. These isolation membranes eventually seal to form an autophagosome with a double-membraned structure. Upon further maturation, the autophagosomes then travel along microtubules to lysosomes, whereupon the outer membranes of autophagosomes fuse with lysosomes to form autolysosomes. Afterwards, single membrane structures release their cargo into lysosomes for degradation by resident hydrolases. Cargo degradation produces small molecules such as amino acids, phospholipids, simple carbohydrates, and fatty acids, which are released subsequently from lysosomes into the cytoplasm for the cell to recycle and reuse^[18]^. Autophagy also serves as one of the main mechanisms to remove damaged organelles such as mitochondria, peroxisomes, lysosomes, and ER^[19]^ and degrade them into small molecules that can serve other cellular functions or lead to resynthesis of organelles or formation of new structures within the cell. There are approximately 20 autophagy-related (ATG) proteins that sequentially participate in the formation of phagophores and their maturation to autophagosomes that fuse with lysosomes to form autolysosomes^[20]^.

### Lipophagy

During starvation conditions, autophagy plays an important role in the utilization of stored lipids that are critical for supplying fuel to generate ATP. Autophagic mobilization of lipid droplets (lipophagy) was initially discovered in hepatocytes and subsequently in other cell types^[21]^. Singh *et al*.^[21]^ showed that autophagic delivery of triglycerides from lipid droplets (LDs) to their hydrolysis in autolysosomes was an essential mechanism for hepatocytes to convert triglycerides stored in LDs to free fatty acids (FFAs) that subsequently underwent oxidation in mitochondria to generate ATP. Additionally, decreased lipid oxidation and increased steatosis occurred in cultured hepatocytes as well as in mouse liver when autophagy was inhibited, suggesting that this was a key mechanism for converting stored triglycerides into FFAs in the liver^[21]^. Another form of autophagy that utilizes heat shock proteins is chaperone-mediated autophagy (CMA), which degrades proteins (perilipin 2 and 3) located at LD membranes to initiate lipophagy^[22]^. During energy stress, AMPK phosphorylates choline kinase α2 (CHKα2) at Ser279, while KAT5 acetylates CHKα2 at Lys247. These modifications activate CHKα2 and phosphorylate LD membrane proteins, perilipin 2 (PLIN2) and 3 (PLIN3). The phosphorylation of PLIN2/3 dissociates PLIN2/3 from LD for CMA-mediated degradation^[23]^.

Lipophagy activation also requires recruitment of autophagy machinery to the LD, a process that is abolished in PLIN3 knockdown cells. PLIN3 indeed directly interacts with autophagy proteins Fip200 and Atg16L^[24]^. IFGGA2 is an immunity-related GTPase which co-localizes with ATGL and LC3B on LDs when animals are fed a high fat diet (HFD). Overexpression of IFGGA2 increases association of LC3B with LDs and decreases lipid content^[25]^. Fasting-induced fibroblast growth factor-21 activates autophagy and lipid degradation through Jumonji-D3 histone demethylase mediated epigenetic changes^[26]^. Natural and pharmacologic ligands for nuclear receptors, such as THR, PPARα, and FXR, as well as β-adrenergic receptors, can also regulate lipophagy^[27–30]^, highlighting the prominent role for endocrine regulation of this cellular process. Fenofibrate, a PPARα agonist, activates lipophagy and reduces hepatic fat accumulation through induction of lysosomal Ca^2+^ release, calcineurin activation, and transcription factor EB (TFEB) and TFE3 dephosphorylation^[31]^. Moreover, we recently showed that a thyromimetic and two PPARα agonists improve hepatosteatosis in the glycogen storage disease GSD1a^[32–34]^. Additionally, natural compounds such as green tea polyphenol or caffeine that stimulate autophagy also reduce hepatosteatosis^[35,36]^. Taken together, these data suggest that inducing and enhancing lipophagy could be novel strategies for treating NAFLD.

### Mitophagy

Mitochondria serve as energy hubs for both oxidation of fatty acids and ATP production through oxidative phosphorylation (OXPHOS). The latter process also generates reactive oxygen species (ROS) that can impair mitochondrial function and integrity to cause mitochondrial depolarization and loss of membrane potential. In mammalian cells, the loss of membrane potential is a strong stimulator that triggers mitophagy^[37]^. Mitophagy is a critical quality control process to eliminate mitochondria damaged by ROS and prevent the initiation of an inflammatory response or apoptosis^[38]^.

Several types of mitophagy are described in the literature^[39]^; however, PINK1-PARKIN-mediated mitophagy is the most extensively characterized mechanism. PINK1, a Ser/Thr kinase, requires a normal mitochondrial membrane potential to be imported into the inner mitochondrial membrane (IMM). Thus, PINK1 serves as depolarization sensor. Loss of membrane potential prevents PINK1’s IMM translocation, leaving it on the outer mitochondrial membrane (OMM) where it is activated subsequently by autophosphorylation^[40–42]^. Active PINK1 phosphorylates several other substrates such as ubiquitin and PARKIN, an E3-ubiquitin ligase that increases the polyubiquitination of OMM proteins^[41]^. Their polyubiquitin chains are recognized by autophagy receptor proteins such as p62 or optneurin that also contain LC3 interaction regions (LIRs) to recruit the key ATG protein, LC3, to promote membrane sequestration of ubiquitinated mitochondrial proteins into autophagosomes.

Several PINK1-PARKIN-independent pathways for mitophagy induction have also been identified^[43]^, including FUN14 domain containing 1 (FUNDC1)-mediated autophagy. FUNDC1 is an OMM protein that contains a LIR facing the cytosol^[44]^. Under normal physiological conditions, FUNDC1 is phosphorylated at Tyr18 and Ser13, which reduces its interaction with LC3. However, during cellular stress such as hypoxia, mitochondrial phosphatase PGAM family member 5 dephosphorylates FUNDC1 at Tyr18 and Ser13^[45]^. Concomitant phosphorylation of FUNDC1 at Ser17 by unc-51-like autophagy activating kinase 1 (ULK1) causes FUNDC1 to become activated and enable it to interact with LC3 to promote mitophagy^[44,46]^.

## Dysregulation of autophagy in NAFLD

**Figure 1:**
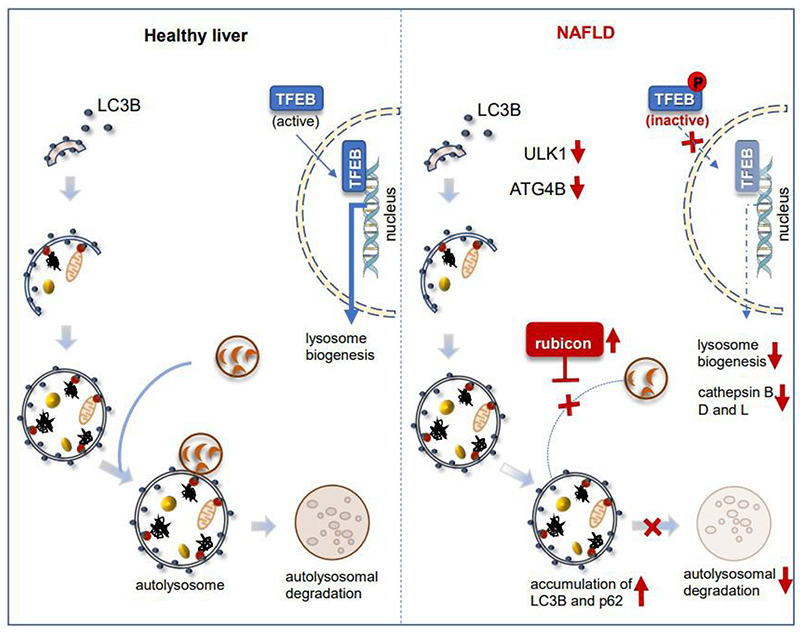
Defects in autophagy in patients with NAFLD. Several steps in autophagy process are affected in patients with NAFLD. First, there is decreased levels of ULK1 and ATG4B. Second, autophagosome/lysosome fusion is decreased due to increased expression of rubicon. Third, decreased nuclear translocation of TFEB, lysosomal biogenesis and lysosomal protease levels, leads to decreased lysosomal degradation and accumulation of LC3B-II and p62. NAFLD: Non-alcoholic fatty liver disease; ULK1: unc-51-like autophagy activating kinase 1; TFEB: transcription factor EB.

Patients with NAFLD have impaired autophagy that typically is manifested by accumulation of both LC3B-II and p62 in hepatic cells^[47]^. Although the increases in LC3B-II and p62 suggest there is a late-stage block in autophagy, multiple steps may be involved [Figure 1]. First, there can be decreased autophagosome/lysosome fusion due to increased expression of rubicon, a negative regulator of autophagosome-lysosome fusion^[48]^, or decreased expression and/or function of the autophagsome/lysosome fusion SNARE complex. Lysosomal function can also be impaired due to decreased levels of lysosomal proteases, leading to decreased lysosomal degradation^[49]^. Additionally, autophagosome biogenesis itself can be impaired preceding or in conjunction with late-stage autophagy block. Zhang *et al*.^[50]^ found that nuclear localization of the master transcriptional regulator for lysosomal biogenesis, TFEB, was negatively associated with steatosis, suggesting that progression of NAFLD from NAFL to NASH can occur through multiple mechanisms.

Rodent models of NAFLD employing high fat content diets recapitulate the main features of NAFLD observed in humans^[21,51]^. Inhibition of autophagy through genetic deletion of key autophagic proteins Atg7^[52]^ and Atg14^[53]^ in hepatocytes further worsened the hepatic phenotype in mouse models of NAFLD. In contrast, restoration of autophagy by overexpression of Atg14^[53]^, TFEB^[50,51]^, or deletion of rubicon^[48]^ improved hepatic steatosis and injury, as well as attenuated cellular stress. Furthermore, deletion of Atg5 in myeloid cells promoted proinflammatory macrophage polarization and increased the inflammatory response in NAFLD^[54]^. Recent data also showed that impaired mitophagy led to formation of megamitochondria that could contribute to liver injury during NAFLD^[55]^. These studies demonstrated that impaired autophagy is one of the main molecular mechanisms that contribute to NAFLD progression. Moreover, restoration of autophagy improves the NASH phenotype and thus could be a novel strategy to treat NAFLD.

Recent studies also identified novel regulators of autophagy in the context of NAFLD. Liver X receptor alpha (LXRα) and sterol regulatory element-binding protein (SREBP)-1C are some of the main transcription factors that regulates lipogenesis. Their expression was upregulated in a rodent model of NAFLD and patients with NAFLD^[56]^. Kim *et al*.^[57]^ showed that LXRα inhibits autophagy through inducing let-7a and miR-34a transcription, which suppress ATG4B and Rab-8b. Furthermore, NAFLD patients had elevated let-7a and miR-34a levels with simultaneous decreases in ATG4B and Rab-8B levels^[57]^. Nguyen *et al*.^[58]^ showed that SREBP-1C-induced miR-216a expression resulted in reduced cystathionine gamma-lyase and hepatic H2S levels, sulfhydration-dependent activation of ULK1, and autophagy flux and lipid degradation. In addition, the expression of ULK1 was downregulated in a mouse model of NAFLD and patients with NAFLD^[59]^. Increased Mir214-3p and decreased Hnf4a expression led to reduced Ulk1 levels in a mouse model of NAFLD^[59]^. Hepatic expression of Acyl-CoA oxidase 1, the enzyme that catalyzes the first step in peroxisomal β-oxidation, was upregulated by HFD feeding; increased hepatic peroxisomal β-oxidation derived acetyl-CoA, raptor acetylation, and activation of mTORC1; and led to decreased autophagy and increased hepatic triglycerides^[60]^.

It is generally believed that lipotoxicity is one of the major triggers for hepatic inflammation and hepatocytes death in patients with NAFLD as disease progresses. Interestingly, emerging evidence also suggests that the stress responses activated in NAFLD also regulate autophagy. Spliced X-box binding protein 1, a key factor that promotes unfolded protein response in response to the ER stress, directly upregulates the expression of TFEB^[61]^. Thioredoxin interacting protein, a key mediator of cellular stress, directly interacts with and positively regulates AMPK phosphorylation to inhibit mTORC1, leading to TFEB activation^[62]^. In contrast, mixed lineage kinase domain-like, a pseudokinase in the necroptotic pathway of programmed cell death, is upregulated by lipotoxic lipid palmitic acid (PA), translocates to autophagosome membrane, and blocks its fusion with lysosome^[63]^. PA also induces stimulator of interferon response cGAMP interactor 1 (STING1), which interacts with several components of the mTORC1 complex and activates mTORC1 to inhibits autophagy^[64]^.

## TH effects on impaired autophagy in NAFLD

Thyroid hormone (TH) is essential for important physiological processes such as development, growth, and metabolism in most higher organisms. Thyroxine (T_4_) and triiodothyronine (T_3_) are the two major circulating THs synthesized and secreted by the thyroid gland. However, T_3_ is the more biologically active form, and its concentration in circulation is much lower than T_4_. Both T_4_ and T_3_ enter cells through transporters that belong to the monocarboxylate transporter 8, organic-anion-transporting polypeptide 1, and the L-type amino acid transporter families^[65,66]^. The deiodinases, deiodinase 1 (DIO1) and deiodinase 2 (DIO_2_), convert intracellular T_4_ to T_3_^[67]^, while DIO3 converts T_3_ to the inactive reverse triiodothyronine (rT_3_). Thyroid hormone receptors (THRs) are members of the nuclear hormone receptor family and are comprised of two major isoforms, THRα1 and THRβ1, encoded by *THRA* and *THRB* genes, respectively. THRα1 is the predominant isoform in bone, cardiac, and skeletal muscle and the gastrointestinal tract, whereas THRβ1 is the predominant isoform in the liver and kidney^[68]^. Intracellular T_3_ binds to nuclear THRs that interact with TH response elements located within the promoters or enhancer regions of target genes and recruit co-activators and RNA polymerase II to regulate transcription. Thus, THRs act as ligand-inducible transcription factors^[66]^.

In the liver, TH regulates genes involved in a diverse range of metabolic pathways, including hepatic lipogenesis, lipid oxidation, cholesterol homeostasis, and gluconeogenesis^[69]^. The transcriptional regulation of hepatic metabolism by TH is reviewed elsewhere^[70–72]^. TH also increases lysosomal acid lipase expression and lysosomal activity^[66,73]^. Sinha *et al*.^[27]^ also showed that TH is a potent stimulator of hepatic lipophagy in cultured hepatic cells and mouse liver. Furthermore, inhibition of autophagy abolished TH-induced hepatic β-oxidation and ketogenesis in mice^[27]^. Thus, TH-induced lipophagy is essential for mobilizing hepatic triglycerides and releasing free fatty acids for mitochondrial β-oxidation. Indeed, acute TH stimulation utilizes lipophagy as the predominant mechanism for hydrolysis of triglycerides from LD, whereas chronic TH stimulation increases hepatic lipase gene expression in addition to autophagy.

T_3_ also promotes hepatic mitophagy, as evidenced by increased engulfment of mitochondria inside autophagosomes and autolysosomes in electron micrographs^[74]^. T_3_ increases the expression and activation of ULK1, which promotes Drp1-mediated mitochondrial fission, FUNDC1 activation and interaction with LC3B, and p62 translocation to mitochondria in hepatic cells^[74,75]^. T_3_-mediated induction of mitophagy is essential for its stimulation of mitochondrial OXPHOS^[74]^. Concurrently, T_3_ also increases mitochondrial biogenesis^[75]^. Thus, T_3_ generates and maintains an intracellular pool of healthy mitochondria to increase mitochondrial activity and β-oxidation of fatty acids by increasing the rates of mitophagy and mitochondrial synthesis, i.e., mitochondrial turnover.

**Figure 2:**
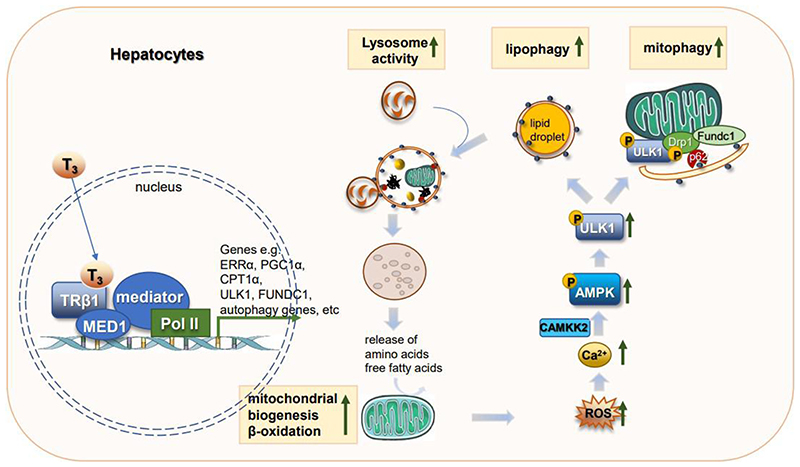
TH effects on hepatic autophagy. TH increases transcription of genes involved in autophagy, mitochondrial biogenesis, and lipid metabolism. Increases in mitochondrial β-oxidation of fatty acids increase ROS which then serve as signaling molecules to activate the CAMKK2-AMPK-ULK1 pathway to stimulate lipophagy. Lipophagy mobilizes lipolysis of triglycerides stored in LDs as free fatty acids that act as fuel for mitochondrial utilization. ROS also activates mitophagy to remove damaged mitochondria. TH: Thyroid hormone; ROS: reactive oxygen species; LDs: lipid droplets; CAMKK2: calcium/calmodulin dependent protein kinase kinase 2; AMPK: AMP-activated protein kinase; ULK1: unc-51 like autophagy activating kinase 1.

The activation of the autophagy/mitophagy pathway by T_3_ is mediated by mitochondrial ROS production. Indeed, in cultured hepatic cells, TH simultaneously increases mitochondrial OXPHOS, mitochondrial ROS generation, and membrane potential. Thus, low levels of T_3_-induced ROS (~40% increase compared to untreated cells) are not toxic but actually serve beneficial roles in cells (hormesis) by acting as signaling molecules to increase cellular Ca^2+^ levels and activate CAMKK2-AMPK signaling^[74]^. Activated AMPK then directly phosphorylates ULK1 to induce phagosome formation for autophagy as well as promotes ULK1 translocation to mitochondria to increase mitophagy [Figure 2]^[76–79]^.

Interestingly, T_3_ also induces the expression of several proteins that contribute to the induction of lipophagy and mitophagy/mitochondrial biogenesis. Estrogen-related receptor α (ERRα) is an orphan nuclear receptor that is transcriptionally induced by T_3_ in an PGC1α-dependent manner. Induction of ERRα by T_3_ is essential for the latter’s effects on mitochondrial biogenesis, fission, mitophagy, and induction of ULK1 expression^[75]^. Mediator complex subunit 1 (MED1) is a major component of the multi-subunit mediator complex that bridges nuclear receptor bound to promoter with RNA polymerase II and serves as a co-activator to increase transcription of target genes regulated by nuclear receptors^[80]^. MED1 regulates transcription of autophagic and mitochondrial genes to increase autophagy/lipophagy, mitochondrial OXPHOS, and β-oxidation of fatty acids^[81]^. Since MED1 directly interacts with THR bound to the promoter region^[82]^, it is necessary for transcriptional regulation of T_3_-induced genes involved in autophagy/lipophagy and mitochondrial OXPHOS^[81]^. T_3_ also increased the expression of β-trophin (C19orf80; also known as ANGPTL8), a protein that is critical for TH-mediated induction of hepatic lipophagy and TAG hydrolysis^[83]^.

Apart from T_3_, other TH metabolites such as T_2_ also mediate hepatic autophagy via induction of TFE3 and TFEB transcription factors to reduce hepatic steatosis in rodent models of NAFLD^[84]^.

## TH effects on inflammasome formation and inflammation

Both TH and mitophagy inhibit the overactivation of NLRP3 inflammasome. NLRP3 inflammasome is an intracellular multiple complex activated in response to pathogen-associated molecular patterns or damaged-associated molecular patters to increase secretion of pro-inflammatory cytokines. NLRP3 inflammasome consists of a sensor protein NLRP3, an adaptor protein ASC, and an effector protein caspase 1. NLRP3 recognition of its activator triggers the assembly of inflammasomes and activation of caspase 1, resulting in the cleavage of pro-interleukin (IL)-1β and pro-IL-18 to matured IL-1β and IL-18. Several signals activate NLRP3 inflammasome, e.g., efflux of potassium ions, lysosomal disruption, metabolic changes, and mitochondrial ROS and mtDNA^[85,86]^. Although inflammasome activation is one of the first lines of host defense, excessive activation can lead to inflammatory diseases such as NAFLD^[87]^. One of the molecular mechanisms that prevent NLRP3 inflammasome overactivation is removal of damaged mitochondria by mitophagy. In macrophages, p62 is upregulated by NF-κB, which is recruited to damaged mitochondria and mediates mitophagy. Loss of this pathway resulted in exacerbated LPS-induced liver inflammation and damage. Thus, the NF-κB-p62-mitophagy pathway serves an intrinsic regulatory mechanism to prevent the overactivation of NLRP3 inflammasomes^[88]^. Autophagy also prevents excessive NLPR3 inflammasome activation through degradation of individual NLRP3 components such as ASC or NLRP3, or the entire NLRP3 inflammasome itself^[89]^.

T_3_ suppresses ischemia-reperfusion-induced hepatic NLRP3 inflammasome activation in an AMPK-dependent manner^[90]^. THRs are expressed in macrophage cell lines^[91]^, and T_3_ negatively regulates NLRP3 inflammasome activation^[92]^. T_3_ enhances the expression of miRNAs miR-30, -133, and -144^[93]^, which inhibit the pro-inflammatory signals by downregulation of fast apoptosis signal ligand^[94]^. T_3_ downregulates the expression miR-31, -155, and -222^[93]^, to increase the gene and protein expression of superoxide dismutase 1 (SOD1) and SOD2^[95]^, which then decrease ROS levels and prevent excessive activation of the NLRP3 inflammasome. In addition, T_3_ downregulates the Toll-like receptor 4 (TLR4)/NF-κB pathway to decrease inflammation^[96,97]^.

## Intrahepatic TH signaling is impaired in patients with NAFLD

Several epidemiological studies have suggested that patients with NAFLD have increased prevalence of overt hypothyroidism and subclinical hypothyroidism^[98,99]^. Likewise, patients with overt and subclinical hypothyroidism have increased prevalence of NAFLD^[100,101]^. In healthy liver, hepatocytes have high DIO1 expression and stromal cells show low expression of DIO3. However, this expression pattern reverses in patients with advanced NASH, as evidenced by decreased DIO1 expression in hepatocytes and increased DIO3 expression in stromal cells^[102]^. These changes in expression of deiodinases during late NASH lower intrahepatic T_3_ concentration, either by decreased conversion from T_4_ to T_3_ or by higher conversion of T_3_ to rT_3_. The circulating rT_3_ level was increased in patients with NASH, providing further evidence supporting the decrease in DIO1 activity during NASH^[102]^. Similar findings were also seen in rats fed a MCD (methionine/choline deficient) diet to induce NASH. Although their circulating T_3_ levels remained unchanged, hepatic DIO1 expression and intrahepatic T_3_ concentration decreased^[103]^. Recently, Bruinstroop showed that, although DIO1 levels were decreased in advanced NASH, there was a compensatory increase in DIO1 expression during hepatosteatosis (early NAFLD) in mice fed a Western diet with fructose^[104]^. Thus, the expression and activity of DIO1 may change as NASH progresses.

There is emerging evidence that impaired TH signaling in hepatic cells is associated with increased fibrosis in NAFLD. Tahara *et al*.^[105]^ showed that, among patients with NAFLD, significantly more patients with subclinical hypothyroidism had higher scores of a non-invasive marker of liver fibrosis, FIB-4 index, than patients with euthyroidism and NAFLD. Manka *et al*.^[106]^ found low free circulating T_3_ was associated with increased liver stiffness measured by transient elastography (Fibroscan). These findings suggest the possibility that decreased circulating T_3_ and/or intrahepatic T_3_ might promote hepatic fibrosis. In this connection, pre-clinical studies suggested TH may have an antifibrotic role since TH and the thyromimetic sobetirome were able to reduce lung fibrosis^[107]^. This anti-fibrotic effect likely was associated with the ability of TH to increase mitochondrial activity and mitophagy since TH did not decrease lung fibrosis in PGC1α- or PINK1-knockout mice^[107]^. Alonso-Merino *et al*.^[108]^ previously showed that TH decreased fibrosis in skin and lung injury models by stimulating THR interaction with TGFβ-induced transcription factors such as SMAD3 and SMAD4. This interaction led to decreased SMAD phosphorylation and its binding to promoters of SMAD target genes involved in fibrosis^[108]^.

## Clinical trials of T4 and thyromemetics in patients with NAFLD/NASH

To determine the clinical efficacy of TH for the treatment for hepatosteatosis, we treated 20 male euthyroid patients with T2DM and steatosis with low-dose T_4_^[103]^. We measured the change in intrahepatic lipid content by proton magnetic spectroscopy (MRI) before and after T_4_ treatment as the primary outcome measure. Patients were treated with daily doses of T_4_ determined by titration to a thyroid-stimulating hormone level between 0.34 and 1.7 mIU/L. After patients were treated with T_4_ for 16 weeks, we found that intrahepatic lipid content decreased by 12% (± SEM, 26%) relative to baseline (*P* = 0.046). Moreover, atrial arrhythmia developed only in one patient. Thus, our study demonstrated low-dose T_4_ was a safe and effective short-term therapy for hepatosteatosis in man [Table 1].

Resmetirom (MGL-3196) is an orally active, selective THRβ1 agonist developed originally to treat obesity and hypercholesterolemia. To assess its efficacy as a treatment for NASH, adult patients with biopsy-proven NASH (fibrosis stages 1-3) and hepatic fat fraction of at least 10% at baseline when assessed by MRI-proton density fat fraction (MRI-PDFF) were treated with MGL-3196 (80 mg once a day) for 36 weeks in a randomized, double-blind, placebo-controlled Phase 2 clinical trial (NCT02912260)^[109]^. The primary endpoint of the study measured the relative change in hepatic fat content in patients receiving MGL-3196 compared with those receiving placebo at Week 12. A secondary endpoint was NASH resolution based upon NASH Activity Score (NAS) of histology at 36 weeks. MGL-3196-treated patients showed a relative reduction of hepatic fat content when compared with placebo-treated patients at Week 12 [-32.9% MGL-3196 (*n* = 78) *vs*. -10.4% placebo (*n* = 38); *P* < 0.0001], and more patients treated with MGL-3196 had a > 2-point NAS reduction in histology of liver biopsy samples than patients treated with placebo (56% MFL-3196 *vs*. 32% placebo; *P* < 0.024). The levels of several serum markers for fibrosis were reduced in patients treated with MGL-3196; however, there were no significant differences observed in the amount of histological liver fibrosis between the MGL-3196 and placebo-treated patients^[109]^. Of note, this study had limitations since it was not adequately powered and the duration of the study may not have been long enough to evaluate changes in fibrosis. Currently, there is a large multicenter Phase 3 study aiming to enroll 2000 participants for evaluating the efficacy of MGL-3196 treatment for NASH (MAESTRO-NASH**/**NCT03900429). The primary outcome measure is resolution of NASH based upon histology of liver biopsies in non-cirrhotic NASH patients with stage 2 or 3 fibrosis after one-year treatment. Study participants will be monitored for long-term adverse outcome events such as all-cause mortality, cirrhosis, and other significant liver-related parameters.

VK2809 (formerly known as MB07811) is a pro-drug that undergoes first-pass hepatic extraction and cytochrome P450 cleavage to generate a negatively charged thyroid THRβ agonist, VK2809A (formerly known as MB07344)^[110]^. VK2809 is highly liver-specific and is rapidly eliminated in the bile^[111]^. VK2809 reduced hepatosteatosis in mouse models of NAFLD^[112]^ and glycogen storage disease type Ia (GSD-Ia), an inherited metabolic liver disease that can develop many similar hepatic features as NAFLD^[33]^. In the mouse model of GSD1a, VK2809 treatment decreased hepatic lipid content through its simultaneous restoration of autophagy, mitochondrial biogenesis, and β-oxidation of fatty acids^[33]^. A Phase 2b, randomized, double-blind, placebo-controlled, multicenter clinical trial is currently underway to assess the efficacy and safety of VK2809 in patients with NASH (VOYAGE/NCT04173065)^[113]^. This study will enroll 337 participants with biopsy-proven NASH and divide them into treatment groups receiving 0, 1.0, 2.5, 5.0, or 10 mg VK2809 orally once a day. The primary outcome measure is the change in fat content (assessed by MRI-PDFF) from baseline to 12 weeks in VK2809- *vs*. placebo-treated subjects. The secondary outcome measure is the percentage of drug- *vs*. placebo-treated subjects who had significant improvement of their NASH based upon histology of liver samples after one year of treatment.

## Conclusion

The pathogenesis and progression of NAFLD is a complex and multifactorial process that involves genetic, epigenetic, and environmental factors. The heterogeneity of NAFLD has created significant challenges for discovering non-invasive biomarkers to diagnose the presence of NASH and/or to prevent or slow down its progression. The identification of homogenous patient groups based upon clinical stage, race, ethnicity, or genotype could improve the discovery and clinical utility of potential markers. They also could provide more specific molecular target(s) that could predict which types of compounds would be more effective for specific subgroups of patients. Nevertheless, recent data strongly suggest that targeting impaired autophagy, mitophagy, and mitochondrial function might be novel intervention strategies for NAFLD in most patients. TH is one example of a compound that can mediate these effects. It not only transcriptionally regulates genes involved in lipid, cholesterol, and glucose metabolism but also induces hepatic autophagy/lipophagy/mitophagy and mitochondrial biogenesis to increase β-oxidation of fatty acids and increase mitochondrial turnover to decrease ROS production and cellular injury. TH and its analogs are effective in treating hepatosteatosis in preclinical models and patients with NAFL. Although several promising preclinical and early clinical studies show that TH or thyromimetics can improve fibrosis in NASH, large-scale placebo-controlled Phase 3 trials need to be undertaken to confirm these findings. Nonetheless, preclinical and clinical studies thus far suggest low-dose of T_4_, THRβ1-selective analogs, or liver-specific thyromimetics could potentially be novel, safe, and effective therapeutic agents against NAFLD/NASH.

## Figures and Tables

**Table 1:** Clinical trials of thyroid hormone, thyroid hormone analogues in patients with NAFLD

Completed studies					
Compound	Treatment regimen	Liver function	Steatosis	NASH resolution	Fibrosis
Levothyroxine^[104]^	20 male patients with type 2 diabetes and steatosis (4 months titrated to a TSH 0.34-1.70 IU/L, median dose 18.75 μg/day	AST, ALT =	Liver fat (H-MRS)?	NA	NA
VK2809^[114]^ meeting abstract (NCT02927184)	24 patients with primary hypercholesterolemia and NAFLD (3 months, 10 mg every day or every other day	AST, ALT ↓	Liver fat (MRI-PDFF) ↓	NA	NA
MGL-3196^[110]^ (NCT02912260)	84 patients with biopsy confirmed NASH (fibrosis stages 1-3) (9 months, 40-80 mg daily)	AST, ALT ↓	Liver fat (MRI-PDFF) ↓	NAS score ↓	Not significant by liver histology
**Recruiting**					
Compound	Treatment regimen	Estimated participants	Primary outcome	Secondary outcome	
VK2809 (NCT04173065)	52-week VK2809 administration (1, 2.5, 5, or 10 mg daily) in patients with biopsy proven NASH with fibrosis	337	Relative change in liver fat content from baseline to Week 12 in subjects treated with VK2809 compared to the change in subjects treated with placebo	Proportion of subjects with resolution of steatohepatitis on overall histopathological reading and no worsening of liver fibrosis on NASH fibrosis score	
MGL-3196 (NCT04197479)	52-week MGL-3196 administration (80 or 100 mg daily) to patients with NAFLD	700	The effect of MGL-3196 *vs*. placebo on the incidence of adverse events	The effect of MGL-3196 *vs*. placebo on the percentage changes in LDL-C, ApoB, hepatic fat fraction, TGs, and PRO-C3	
MGL-3196 (NCT04951219)	52-week MGL-3196 administration (80 or 100 mg daily) to patients with NAFLD	1000	The effect of MGL-3196 *vs*. placebo on the incidence of adverse events	Percentage change in the hepatic fat fraction from baseline at week 16 and 52; percentage change in LDLC from baseline at week 28	
MGL-3196 (NCT03900429)	52-week MGL-3196 administration (80 or 100 mg daily) to patients with NASH and fibrosis	2000	The effect of MGL-3196 *vs*. placebo o achieve NASH resolution on liver histology in non-cirrhotic NASH patients with stage 2 or 3 fibrosis at week 52; Composite long-term outcome events composed of all-cause mortality, cirrhosis, and other significant liver-related events (up to 54 months)	The effect of MGL-3196 *vs*. placebo on the percentage changes in LDL-C from baseline at week 24; The effect of MGL-3196 *vs*. placebo to achieve improvement in fibrosis on liver histology in non-cirrhotic NASH patients with stage 2 or 3 fibrosis from baseline at week 52	

H-MRS: Magnetic resonance spectroscopy; LDL-C: low-density lipoprotein cholesterol; MRI-PDFF: magnetic resonance imaging proton density fat fraction; PRO-C3: N-terminal type III collagen propeptide; NAFLD: nonalcoholic fatty liver disease; NASH: nonalcoholic steaheatitis.

## Data Availability

Not applicable.
